# Polypathology as a Marker of Care Complexity in Hospitalized Adults with Multimorbidity in México

**DOI:** 10.3390/jcm15218722

**Published:** 2026-11-08

**Authors:** Kevin Javier Arellano-Arteaga, Javier Israel Campechano-Aguirre, Elvira Miramontes-Luna, Martin Bedolla-Barajas, Jorge Andrade-Sierra, Francisco Gerardo Yanowsky-Escatell, Pablo Maggiani-Aguilera

**Affiliations:** 1 Departamento de Clínicas Médicas, Centro Universitario de Ciencias de la Salud, Universidad de Guadalajara, Guadalajara 44340, Jalisco, Mexico; javier.camp.96@gmail.com (J.I.C.-A.); miramontesle@hotmail.com (E.M.-L.); 2 Departamento de Medicina Interna, Nuevo Hospital Civil de Guadalajara “Dr. Juan I. Menchaca”, Guadalajara 44340, Jalisco, Mexico; drmbedbar@gmail.com (M.B.-B.); fyanowsky@hotmail.com (F.G.Y.-E.); 3 Instituto de Terapéutica Experimental y Clínica, Centro Universitario de Ciencias de la Salud, Universidad de Guadalajara, Guadalajara 44340, Jalisco, Mexico; jorge.andrade@academicos.udg.mx; 4 Departamento de Medicina Interna, Hospital General de Mazatlán, Mazatlán 82267, Sinaloa, Mexico; maggiani@gmail.com

**Keywords:** polypathology, multimorbidity, Charlson index, internal medicine, hospital mortality, polypharmacy, uninsured population

## Abstract

**Background/Objectives:** The concept of the polypathological patient was developed in Spain, in older populations, and has not been described in México. We analyzed its frequency, profile, relationship with care burden, and phenotypes in hospitalized adults with multimorbidity and without social security coverage. **Methods:** This was a retrospective single-center cohort of patients with multimorbidity admitted to the Internal Medicine department of a public university hospital in Guadalajara, Jalisco, Mexico (2023–2024). Polypathology was defined using the 2018 Andalusian criteria. Logistic regression adjusted for age, sex, and the Charlson index, and latent class analysis were used. **Results:** Of 1108 patients with multimorbidity, 702 (63.4%) were polypathological; their mean age was 61.5 years, 43.6% were younger than 60, and 51.7% were men. Each additional category was associated with polypharmacy (OR 1.58; 95% CI 1.35–1.85) and prior hospitalizations (1.63; 1.36–1.95) at admission and with discharge at maximum therapeutic benefit (1.63; 1.23–2.18), also after adjustment for the Charlson index, but not with death during the index admission (0.94; 0.77–1.16). Seven phenotypes were identified, which differed in polypharmacy and prior hospitalizations but not in mortality. **Conclusions:** In this cohort, polypathology was frequent, affected patients about 15 years younger than in Spanish cohorts, although the cohorts differ in population and setting, and was associated with markers of care burden, with no clear association with in-hospital mortality. Prospective multicenter studies are needed to confirm these findings.

## 1. Introduction

Multimorbidity, defined as the coexistence of two or more chronic diseases, affects 37.2% of community-dwelling adults worldwide and 45.7% in South America [1]. In Latin America and the Caribbean, the pooled prevalence is 43% [2]. In México, the Continuous National Health and Nutrition Survey 2021–2024 reported a prevalence of type 2 diabetes of 17.0% and of arterial hypertension of 29.1% in adults, with poor control of both [3,4].

The definition of multimorbidity has known limitations. Published prevalences range from 12.9% to 95.1% depending on the number and type of diseases considered [5]. In addition, a simple count of diagnoses does not distinguish between disease burden, severity, and complexity of management, which are distinct dimensions [6]. In the hospital setting, the problem becomes more pronounced. In a Swiss series of more than two million medical discharges, 79.7% of patients had multimorbidity [7]. When almost all patients have it, the concept no longer separates those who need a different kind of care.

The concept of the polypathological patient was proposed in Andalusia to address this problem [8]. It requires symptomatic chronic diseases in at least two of eight clinical categories (A to H), or in one category with a complexity criterion in the 2018 version, and it incorporates severity criteria, such as functional class in heart failure or the degree of cognitive impairment. In its initial validation, polypathological patients admitted to Internal Medicine had a mean age of 75 years and a mortality of 19.3%, compared with 6.1% in the rest [9]. A specific index, the PROFUND index, was developed to estimate their prognosis and validated for mortality at 12 months and at four years [10,11].

Almost all the evidence on polypathology comes from Spain, from aging populations with universal health coverage. In Latin America, we found only an Argentine cohort of 111 patients [12] and a Venezuelan series of 79 patients [13], and we identified no studies in Mexico. This gap is relevant because the Mexican population without social security coverage has late diagnosis, poor control of chronic diseases, and fragmented access to care [3,4], factors that may bring forward the age at which diseases accumulate. Nor do we know whether polypathology provides information different from that of an established comorbidity index, such as the Charlson index [14]. Both instruments share much of their content but differ in purpose; the Charlson index weights diagnose according to their association with mortality, whereas the polypathology criteria require symptomatic disease or functional limitation and include conditions that the Charlson index does not capture, such as chronic anemia or disabling osteoarticular disease. In a Spanish continuity-of-care unit linking primary care and internal medicine, polypathology was associated with hospital admission more strongly than the Charlson index [15], but we are not aware of studies comparing both instruments against markers of care complexity in hospitalized patients.

The objectives of this study were to describe the frequency and clinical profile of polypathology within multimorbidity, comparing polypathological patients with those who have multimorbidity without polypathology; to analyze whether polypathology and the number of categories are associated with markers of care complexity and with hospital outcomes, including in-hospital mortality, independently of age, sex, and the Charlson index; and to identify clinical phenotypes within the polypathological population.

## 2. Materials and Methods

### 2.1. Design

We conducted a retrospective single-center cohort study in the Internal Medicine department of the Nuevo Hospital Civil de Guadalajara “Dr. Juan I. Menchaca”, a university hospital affiliated with the Universidad de Guadalajara. The hospital mainly serves a population not affiliated with the Mexican Social Security Institute, the Institute for Social Security and Services for State Workers, or private insurance. This report follows the STROBE statement for observational studies [16].

Consecutive admissions captured in a census registry of the department from April 2023 to May 2024 were included.

### 2.2. Population

A total of 2455 admissions to the department were recorded during the period, corresponding to 1966 unique patients identified by the medical record number. Each patient was analyzed once, with the clinical data of the first admission, and the 489 subsequent admissions were used to define readmission during the period (during the follow-up period, 247 patients had at least one hospital readmission, accounting for a total of 348 readmission events). Of the 1966 patients, 1111 had multimorbidity and 855 had one or no chronic disease. Three patients younger than 18 years were excluded. The final sample comprised 1108 patients (Figure 1).

The eligibility criteria were as follows:

- Inclusion: adults admitted to the Internal Medicine department during the study period with multimorbidity documented in the medical record at admission;
- Exclusion: age younger than 18 years.

In patients with more than one admission during the period, only the first (index) admission was analyzed for the clinical variables.

### 2.3. Definitions

**Multimorbidity.** Simultaneous presence of two or more chronic diseases documented in the medical record at admission [5]. Chronic diseases from eleven groups were considered: cardiovascular, respiratory, neurological, endocrine and metabolic, gastrointestinal, renal and urinary, chronic infectious, musculoskeletal and immunological, hematological and oncological, psychiatric, and a residual group of chronic diseases not classifiable in the previous groups.

**Polypathology.** We applied the clinical categories of the 2018 Integrated Care Process for polypathological patients (*Proceso Asistencial Integrado*, *PAI*) [8]: A, heart failure with NYHA class II dyspnea in a stable situation, or ischemic heart disease; B, vasculitis or systemic autoimmune disease, or chronic kidney disease; C, chronic respiratory disease with mMRC grade 2 dyspnea, FEV1 < 70%, or baseline oxygen saturation ≤ 90%; D, inflammatory bowel disease or chronic liver disease with hepatocellular failure or portal hypertension; E, stroke, neurological disease with permanent motor deficit limiting basic activities, or at least moderate cognitive impairment; F, symptomatic peripheral artery disease, or diabetes with proliferative retinopathy or symptomatic neuropathy; G, chronic anemia (hemoglobin < 10 g/dL in two determinations more than three months apart) due to digestive losses or acquired hemopathy not amenable to curative treatment, or active solid or hematological neoplasm not amenable to treatment with curative intent; H, chronic osteoarticular disease limiting bed-to-chair transfer, or osteoporotic hip fracture (Table S1). Patients with two or more categories were classified as polypathological. The 2018 Integrated Care Process also includes patients who meet a single category together with a complexity criterion (e.g., extreme polypharmacy, two or more admissions in 12 months, socio-familial risk, or delirium); this alternative route was not applied because several complexity criteria were not systematically recorded, so polypathology was defined only by the classic criterion of two or more categories. Because the criteria were applied only to patients with multimorbidity, every polypathological patient had multimorbidity; the comparison group consisted of patients with multimorbidity who did not meet the polypathology criteria. The number of categories met (0 to 8) was recorded.

All categories were assigned using the information recorded in the medical record. The Integrated Care Process defines subcategories E2 and H1 by a Barthel index below 60 and E3 by a standardized cognitive test. In the department, the functional scale recorded is the Katz index, which has no validated equivalent cutoff and was missing in half of the patients, and cognitive tests were not applied systematically. Therefore, subcategories E2, E3, and H1 were assigned from the clinical description recorded in the medical record of dependence for basic activities, inability to transfer from bed to chair, or documented cognitive impairment.

Data were collected from the medical records by one investigator with a standardized census form and verified by a second investigator for all patients. Polypathology categories were abstracted from the information of the index admission before the hospital outcome was reviewed; because the outcome is documented in the same record, abstractors were not formally blinded to it. A criterion was considered met only when the required information was documented; otherwise, it was coded as not met. Subcategory B2 required a documented diagnosis of chronic kidney disease, not an isolated creatinine value, and subcategory G1 required hemoglobin < 10 g/dL in two determinations more than three months apart, verified in the hospital laboratory records, which are available because patients without social security coverage are consistently treated at this hospital.

**Charlson index.** The original index [14] was calculated from its 16 weighted components, without age points. Separately, the age-adjusted index [17] was calculated, which adds one point for each decade from age 50 years (maximum four). Hereafter, “Charlson index” refers to the original index; the age-adjusted index is used only where stated (Table 1, Tables S3 and S6, Figure S1, and Section 2.5 and Section 4.4).

**Other variables.** Functional status was assessed with the Katz index [18], classified as independent (A) or dependent (B to G). Polypharmacy was defined as five or more drugs in chronic use for chronic diseases at admission, excluding as-needed medications, and severe polypharmacy as ten or more [19]. Hospitalizations in the previous 12 months in the Internal Medicine department itself were obtained from the medical record at the index admission (0–1 versus 2 or more). Body mass index was classified according to the World Health Organization categories. Serum hemoglobin and creatinine were taken as the first value recorded within the first 24 h of admission; body mass index, hemoglobin, and creatinine were available for all patients. In patients of the hepatic-hematologic phenotype, the etiology of liver disease, alcohol consumption, and the cause of anemia recorded in the medical record were also collected.

**Markers of care complexity and outcomes.** Polypharmacy, two or more prior hospitalizations, and functional dependence were recorded at the index admission and were analyzed as cross-sectional markers of care complexity, not as outcomes. The primary hospital outcome was death during the index admission, which could be ascertained in all patients because those readmitted had necessarily survived that admission. The census recorded a single type of discharge per patient, corresponding to the last discharge of the period. Therefore, discharge at maximum therapeutic benefit, death during any admission of the period, the composite of both, and, on an exploratory basis, readmission during the period (one or more admissions after the first, identified by the medical record number) were analyzed as period outcomes, with adjustment for follow-up time. Discharge at maximum therapeutic benefit was defined as the discharge of patients for whom no further disease-modifying treatment options were available, generally to continue end-of-life care at home. These decisions were made jointly by the treating internal medicine team and the palliative care service, considering the patient’s clinical condition, prognosis, available therapeutic options, family circumstances, and resources available to support care at home.

### 2.4. Sample Size

No prospective sample size calculation was performed, because all eligible patients were consecutively included during the study period. The final sample comprised 1108 patients, with 139 deaths during the index admission. The adequacy of the sample for the mortality analysis was assessed by considering the number of available events, the complexity of the multivariable model, and the precision of the estimates, expressed as 95% confidence intervals. With 702 and 406 patients, the study had 80% power (two-sided α = 0.05) to detect an increase in mortality during the index admission from 10.8% to 16.8% (OR ≈ 1.7), but not weaker associations.

### 2.5. Statistical Analysis

Continuous variables are described as mean ± standard deviation or median (interquartile range) and were compared with Welch’s *t* test or the Mann–Whitney U test. Categorical variables are described as frequencies and percentages and were compared with Pearson’s χ^2^ test (with Yates’ correction in 2 × 2 tables) or with Fisher’s exact test when more than 20% of the expected frequencies were below 5 (with Monte Carlo simulation in tables larger than 2 × 2). Correlations were assessed with Spearman’s coefficient.

The relationship between the number of polypathology categories (0, 1, 2, 3, and 4 or more) and each complexity marker and outcome was analyzed with logistic regression, entering the number of categories as a continuous variable and adjusting for age (per decade) and sex and, for period outcomes, for the logarithm of the months from the index admission to the end of the period, to account for the different opportunity to be readmitted or to die according to the date of the first admission; because the census recorded only the last discharge of the period, a time-to-event analysis was not possible. To verify that the gradient did not merely reflect comorbidity burden, the analysis was repeated with the original Charlson index as an additional covariate, and the area under the ROC curve (AUC) of the number of categories was calculated for each marker. To examine nonlinear patterns, the number of categories was also entered as a categorical variable (0 as reference) and with a quadratic term. For each marker and outcome, the effect of polypathology adjusted for the original Charlson index was also estimated.

Mortality during the index admission was described by strata of the original Charlson index (0–1, 2–3, 4–5, 6–7, and ≥8 points). The primary estimate of the association between polypathology and mortality came from a logistic model adjusted for age, sex, and the original Charlson index (continuous); a secondary model, specified a priori, also included polypharmacy and prior hospitalizations. With 139 deaths, the primary and secondary models had about 35 and 20 events per parameter, respectively. Collinearity was assessed with the variance inflation factor. To quantify the effect of including the age-adjusted Charlson index together with age, as is frequently done in the literature, a model with the age-adjusted Charlson index ≥ 4 and continuous age was also fitted. As a sensitivity analysis, the models were repeated for death during any admission of the period, with adjustment for follow-up time. Discharge at maximum benefit was also analyzed in the 861 patients without readmission, in whom the recorded discharge corresponds to the index admission, and readmission in the 969 survivors of the index admission. Underweight and voluntary discharge were compared between groups with logistic regression adjusted for age and sex and, for voluntary discharge, also for follow-up time.

As a complementary analysis, discrimination for death during the index admission was measured with the AUC and its confidence intervals by the DeLong method, and the added value of polypathology over a model with age, sex, and the Charlson index was assessed with the DeLong test for correlated curves, the continuous net reclassification index, and the integrated discrimination improvement [20,21,22], with bootstrap confidence intervals (500 replicates). Calibration was assessed with the Hosmer–Lemeshow test.

To identify polypathology phenotypes, latent class analysis was applied to polypathological patients, with the eight categories (A to H) as binary indicators. Models with two to eight classes were fitted by maximum likelihood with the expectation–maximization algorithm, with 300 sets of random starting values per model to avoid local solutions. The number of classes was chosen using the Bayesian information criterion (BIC), relative entropy, mean posterior probability of membership, and clinical interpretability [23,24]. Each patient was assigned to the class with the highest posterior probability. Phenotypes were compared with the Kruskal–Wallis or χ^2^ tests, and with likelihood ratio tests in logistic models adjusted for age and sex, and for age, sex, and the Charlson index. In the analyses of period outcomes, the logarithm of the months elapsed from the first admission to the end of the period was added as a covariate.

The Katz index, the only variable with missing data (not recorded in 50.4% of patients), was neither imputed nor included in the main model. The characteristics of patients with and without a recorded Katz index were compared, and in the 550 patients with a record, a mortality model was fitted that added functional dependence (Katz B–G) to age, sex, the original Charlson index, and polypathology. A two-sided *p* value < 0.05 was considered significant. No correction for multiple comparisons was applied, except for the comparison of the eight categories (Table S2); the phenotype analyses and the period outcomes are considered exploratory, and *p* values between 0.01 and 0.05 should be interpreted with caution.

Analyses were performed in R 4.6.1 [25], with the packages pROC 1.19.1 for the AUCs, their confidence intervals, and the DeLong test [26]; nricens 1.6 for the net reclassification index [27]; boot 1.3-32 for the bootstrap confidence interval of the integrated discrimination improvement [28]; ResourceSelection 0.3-6 for the Hosmer–Lemeshow test [29]; and poLCA 1.6.0.2 for latent class analysis [30]. The reported values correspond to R. The code is available from the corresponding author on request.

### 2.6. Ethical Aspects

The study was conducted in accordance with the Declaration of Helsinki and the Regulations of the General Health Law on Health Research. It was approved by the hospital’s Research Ethics Committee under approval number CEI-00048. Because it was a retrospective study without patient contact, the committee waived informed consent. Data were analyzed without personal identifiers.

## 3. Results

### 3.1. Characteristics of the Population

Of the 1108 patients with multimorbidity, 702 (63.4%) met the polypathology criteria, equivalent to 35.7% of the 1966 patients admitted during the period. The mean age of polypathological patients was 61.5 ± 15.8 years; 306 (43.6%) were younger than 60 years and 155 (22.1%) younger than 50 (Table 1; Figure 2). Compared with patients with multimorbidity without polypathology, they were older and had a greater comorbidity burden (median original Charlson index 4 versus 2), more polypharmacy, more prior hospitalizations, more functional dependence, and less underweight. There were no differences in sex distribution (Table 1) or length of hospital stay (Table 2).

Multimorbidity without polypathology had a distinct profile. These patients more frequently had chronic infectious diseases (12.6% versus 5.3%; 22 with AIDS versus 5), hemato-oncological diseases (20.2% versus 14.4%), and psychiatric diseases (6.7% versus 3.1%), and less frequently cardiovascular (66.5% versus 82.5%), endocrine (40.9% versus 69.4%), and respiratory diseases (6.9% versus 14.1%) (Table 1). Underweight and voluntary discharge were more frequent without polypathology (ORs for polypathology of 0.51; 95% CI 0.35–0.75, adjusted for age and sex, and 0.40; 0.20–0.82, additionally adjusted for follow-up time, respectively). Twenty-nine patients without polypathology had a Charlson index of 5 or more; their mean age was 42.6 years, and 22 of them had AIDS. Conversely, 99 polypathological patients had a Charlson index of 2 or less.

### 3.2. Polypathology Categories

Of the 702 polypathological patients, 480 (68.4%) met two categories and 222 (31.6%) three or more. The most frequent categories were F (diabetes with retinopathy or neuropathy, or peripheral artery disease; 61.0%), A (heart disease, predominantly heart failure; 53.6%), and B (predominantly chronic kidney disease; 44.0%). Mean age varied markedly between categories: 54.3 years in D (predominantly chronic liver disease), 57.4 in G (chronic anemia or neoplasia), and 58.6 in B, versus 64.4 in A and 66.8 in E (Table S2). The youngest combination was that of exclusively D and G, with 56 patients and a mean age of 49.4 years. Among polypathological patients, mortality during the index admission did not differ clearly between categories after adjustment for age and sex; categories E (OR 1.65; 95% CI 1.00–2.70) and C (OR 0.45; 0.21–0.97) showed borderline associations of opposite direction that would not remain significant after correction for eight comparisons (Table S2). The triad of heart failure, chronic kidney disease, and diabetes with microvascular damage (A1 + B2 + F2) was found in 66 patients (9.4%), with a mortality of 13.6% versus 13.5% in the remaining polypathological patients.

### 3.3. Number of Categories, Markers of Care Complexity, and Outcomes

The prevalence of polypharmacy and prior hospitalizations at admission and the frequency of discharge at maximum therapeutic benefit increased with each additional category (Table 3; Figure 3). Polypharmacy rose from 14.8% in patients with no category to 44.0% in those meeting three and 41.4% in those meeting four or more. Mortality during the index admission did not follow this pattern: it was 8.2%, 11.3%, 14.6%, 12.4%, and 3.4% in the five groups (OR per category 0.94; 0.77–1.16). A linear OR does not adequately describe this inverted U-shaped pattern (quadratic term, *p* = 0.029); with the number of categories as a categorical variable, no level differed significantly from zero categories (Table S8).

When polypathology was analyzed as a dichotomous variable, only its association with polypharmacy persisted after adjustment for the Charlson index; the associations with prior hospitalizations and with discharge at maximum benefit lost significance (Table S9).

In contrast, the gradient by number of categories persisted after adding the Charlson index, despite the correlation between both scales (Spearman’s ρ = 0.64): OR per category of 1.49 (1.23–1.81) for polypharmacy, 1.34 (1.08–1.66) for prior hospitalizations, and 1.46 (1.03–2.08) for discharge at maximum benefit (Table 3); in the 861 patients without readmission, in whom the recorded discharge corresponds to the index admission, the OR for the latter was 1.79 (1.28–2.51), and 1.45 (0.97–2.16) with the Charlson index. With the Charlson index in the model, the OR for death during the index admission was 0.74 (0.58–0.95; *p* = 0.018). The discrimination of the number of categories was modest and similar to that of the Charlson index (AUC 0.57 to 0.62; Table S9), and length of stay did not vary with the number of categories (ρ = 0.03; *p* = 0.35).

For the composite outcome of death during any admission or discharge at maximum benefit, polypathology showed an OR adjusted for age, sex, and follow-up time of 1.34 (0.98–1.84; *p* = 0.069), which was nullified after adjustment for the Charlson index (OR 1.03; 0.72–1.47). Overall, 22.3% of patients had at least one readmission during the period, with no difference between patients with and without polypathology (22.2% versus 22.4%; adjusted OR 1.08; 0.79–1.47) and no gradient by number of categories (OR 1.03; 0.87–1.22), also when the analysis was restricted to the 969 survivors of the index admission (OR 1.10; 0.80–1.50).

### 3.4. In-Hospital Mortality

A total of 139 patients (12.5%) died during the index admission, and 43 more died during a readmission in the period (182 deaths in any admission; 16.4%). Mortality during the index admission increased with the original Charlson index up to the 6–7-point stratum (7.5%, 11.0%, 15.8%, and 17.0%) and was 13.0% in the ≥8-point stratum, which included only 23 patients (Table 4; *p* for overall comparison = 0.043). The proportion of polypathological patients reached 93.8% in the 4–5-point stratum and decreased in the higher strata, in which 22 of the 26 patients without polypathology had AIDS, a diagnosis that weighs 6 points in the Charlson index but is not part of the polypathology categories. Mortality was 17.3% in patients aged 60 years or older and 7.7% in those younger than 60 (*p* < 0.001).

Mortality during the index admission was 13.5% in polypathological patients and 10.8% in the others (crude OR 1.29; 95% CI 0.88–1.88; *p* = 0.19). In the primary model, age (OR 1.32 per decade) and the original Charlson index (OR 1.15 per point) were independently associated with death, whereas the association with male sex was not significant (OR 1.37; 0.94–1.98). Polypathology showed no clear association with death (OR 0.84; 0.54–1.30), although the confidence interval does not exclude a moderate association in either direction (Table 5). Mortality was 14.0% in men and 11.0% in women (*p* = 0.15). Variance inflation factors were below 1.4 for all variables.

The age-adjusted Charlson index was correlated with age (r = 0.62, versus 0.06 for the original index). With the age-adjusted Charlson index ≥ 4 and age in the same model, the OR for the index was 2.26 (1.25–4.11) and that for age decreased from 1.31 to 1.18 per decade, which is consistent with age being counted twice.

In the secondary model, which added polypharmacy and prior hospitalizations, the OR for polypathology was 0.90 (0.58–1.40). Patients with two or more prior hospitalizations had lower mortality during the index admission (OR 0.30; 0.15–0.59): 55.1% of them were readmitted during the period, versus 13.9% of the other patients, and 67.7% of their deaths occurred during readmissions, versus 14.6% in the other patients (*p* < 0.001). For death during any admission of the period, with adjustment for follow-up time, the OR for polypathology was 0.90 (0.60–1.33) and that for male sex 1.66 (1.19–2.32) in the primary model, and that for prior hospitalizations 0.88 (0.57–1.38) in the secondary model (Table S8).

The 558 patients without a recorded Katz index were younger than the 550 with a record (53.5 ± 16.9 versus 63.5 ± 16.8 years; *p* < 0.001) and included a higher proportion of men (56.3% versus 46.7%), but they had the same median Charlson index (3 in both groups) and a similar proportion of polypathology (63.6% versus 63.1%), and somewhat lower mortality during the index admission (10.6% versus 14.5%; *p* = 0.057) (Table S4). In the 550 patients with a recorded Katz index (80 deaths), functional dependence showed a nonsignificant association with death (OR 1.55; 95% CI 0.90–2.65) after adjustment for age, sex, and the Charlson index, and polypathology remained unassociated (OR 0.74; 0.41–1.33).

The discrimination of polypathology for death during the index admission was low (AUC 0.53; 95% CI 0.49–0.57), lower than that of age (0.62) and of the original Charlson index (0.57), and adding it to a model with age, sex, and the Charlson index did not change the AUC (*p* = 0.52), the integrated discrimination improvement (0.0008; 95% CI −0.00005 to 0.0088), or reclassification (Table S6; Figure S1).

### 3.5. Polypathology Phenotypes 

The seven-class model had the lowest BIC (5253.2), an entropy of 0.97, and mean posterior probabilities of membership of 0.95 to 1.00 (Table S5). The seven classes corresponded to clinically recognizable phenotypes (Table 6 and Table S7; Figure 4). The most frequent was the renal-diabetic phenotype (166 patients; 23.6%), in which all patients had chronic kidney disease and diabetes with microvascular damage or peripheral artery disease. The second was the hepatic-hematologic phenotype (144; 20.5%), defined by chronic liver disease (category D) and chronic anemia or active neoplasia (category G), with estimated probabilities of 72% and 90%, with a mean age of 55.9 years and the highest proportion of men (66.0%). In the 107 patients of this phenotype with chronic liver disease, the etiology recorded in the medical record was alcohol-related in 69 (64.5%), metabolic in 19 (17.8%), and hepatitis C virus in 7 (6.5%); alcohol predominated in men (58 of 76; 76.3%) and metabolic disease in women (13 of 31; 41.9%). In the 119 patients with chronic anemia (G1), the cause was digestive loss in 105 (88.2%), 46 of them due to varices or portal hypertension. These were followed by the neurological (123; 17.5%), cardiometabolic (97; 13.8%), cardiorenal (78; 11.1%), cardiorespiratory (64; 9.1%), and osteoarticular (30; 4.3%) phenotypes, the last comprising all patients with category H.

The phenotypes differed in age, sex, and Charlson index (*p* < 0.001 in all three comparisons). Polypharmacy was more frequent in the cardiorenal (43.6%), cardiorespiratory (42.2%), and renal-diabetic (41.6%) phenotypes, and less frequent in the hepatic-hematologic phenotype (19.4%). Prior hospitalizations were concentrated in the cardiorenal (37.2%), renal-diabetic (34.3%), and hepatic-hematologic (27.8%) phenotypes, versus 9.8% to 13.3% in the others. Both differences persisted after adjustment for age, sex, and the Charlson index (*p* < 0.001). Discharge at maximum therapeutic benefit ranged from 2.1% to 10.8% (*p* = 0.049 with adjustment for age, sex, and follow-up time; *p* = 0.10 after adding the Charlson index). Mortality during the index admission did not differ between phenotypes (9.0% to 20.3%; *p* = 0.23 with adjustment for age and sex).

Readmission during the period varied between phenotypes, from 13.8% in the neurological to 30.8% in the cardiorenal phenotype (*p* = 0.051 with adjustment for age, sex, and follow-up time; *p* = 0.046 after adding the Charlson index). The three phenotypes with the most prior hospitalizations, cardiorenal, hepatic-hematologic, and renal-diabetic, were also those with the most readmissions (28.4%, versus 14.6% in the other four phenotypes and 22.4% in multimorbidity without polypathology). These three phenotypes were also the youngest (mean age 55.7 to 58.2 years) and included 77.4% of polypathological patients younger than 50 years, versus 49.0% of those aged 50 years or older (*p* < 0.001).

## 4. Discussion

In this cohort of hospitalized adults without social security coverage, two of every three patients with multimorbidity met the polypathology criteria, and four in ten of them were younger than 60 years. The number of categories was associated in a graded manner with polypharmacy and prior hospitalizations at admission and with discharge at maximum therapeutic benefit, and the association persisted after adjustment for the Charlson index. In contrast, polypathology showed no clear association with mortality during the index admission. To our knowledge, this is the first study to describe polypathology in México and the largest Latin American series published.

### 4.1. Earlier Polypathology

The age difference from the Spanish cohorts is the most striking finding. Polypathological patients in the original series had a mean age of 75 years [9], those in a later Spanish multicenter cohort 77.3 years [31], those in the Argentine cohort 75.8 years [12], and those in the Venezuelan series 66.2 years [13]. Our patients were 14 to 16 years younger than those in the Spanish and Argentine series and five years younger than those in the Venezuelan series (Figure 2); their mortality during the index admission (13.5%) was somewhat lower than that reported in those series (17–19%) [9,12,13], although differences in outcome definitions limit this comparison. Comparisons between cohorts should be interpreted with caution, as they differ in country, period, health system, selection criteria, and type of hospital, and our entire cohort with multimorbidity was young (mean age 58.5 years), so part of the difference reflects the population served by the hospital and not only an earlier onset of polypathology.

The category profile may partly explain this difference. Diabetes with microvascular damage, heart disease, and chronic kidney disease predominated, diseases whose progression depends largely on metabolic and blood pressure control. In México, only one-third of adults with diagnosed diabetes have adequate glycemic control [3], and the population without social security coverage has less access to continuous care. The categories with the lowest mean age (D, G, and B) also suggest a contribution of chronic liver disease and of renal and autoimmune diseases in young adults, which the phenotype analysis makes more visible (Section 4.3).

### 4.2. Polypathology as a Marker of Care Complexity

The gradient between the number of categories and the complexity markers was the most consistent result of the study. Because polypharmacy and prior hospitalizations were recorded at admission, these are cross-sectional associations that describe the care burden patients bring to the hospital, not consequences of polypathology. Each additional category was associated with an increase of about 60% in the odds of polypharmacy, repeated hospitalizations, and discharge at maximum therapeutic benefit. That the gradient persisted after adjustment for the Charlson index, clearly for polypharmacy and prior hospitalizations and at the limit of significance for discharge at maximum benefit, despite the correlation between both instruments, suggests that the number of categories captures information not fully contained in the Charlson index; the Charlson index summarizes comorbidity burden with weights derived from mortality, whereas the polypathology criteria require symptomatic disease and functional limitation, and therefore may better reflect care needs [6]. A Spanish study found a concordant result, in which polypathology was associated with hospital admission independently of the Charlson index and more strongly (adjusted OR 2.51 versus 1.81) [15].

Three caveats qualify this conclusion. First, it applies only to the number of categories: when polypathology was analyzed as a dichotomous variable, its associations with prior hospitalizations and with discharge at maximum benefit lost significance after adjustment for the Charlson index, and only the association with polypharmacy persisted. Second, discrimination was modest (AUC 0.57 to 0.62) and similar to that of the Charlson index (0.59 to 0.62), so the number of categories serves to characterize the patient’s care needs, not as a screening test to decide who is offered a different model of care. Third, part of the association with polypharmacy is expected, because each category adds chronic diseases that require treatment; adjustment for the Charlson index attenuates this circularity but does not eliminate it. The complexity captured by polypathology is therefore that of chronic care and not that of the acute episode; the number of categories was not associated with length of stay or, significantly, with functional dependence.

This interpretation is consistent with the purpose for which the concept was created, namely to identify patients who need a comprehensive and continuous model of care [8]. In Spain, a multidisciplinary intervention aimed at polypathological patients reduced hospital admissions from 1.52 to 0.82 per patient in one year [32]. Our data indicate that, in a Mexican public hospital, this group is large and relatively young, and suggest that the number of categories may help to grade the intensity of the care it needs, a hypothesis that requires prospective evaluation.

The comparison group was not made up of less severely ill patients but of younger patients with more chronic infection, AIDS, cancer, psychiatric disease, and underweight, conditions that the polypathology criteria do not contemplate because they were designed for chronic organic disease with functional limitation [8]. Hospital multimorbidity thus encompasses at least two complexity profiles, one organic and functional, captured by polypathology, and another marked by chronic infection, malnutrition, and probably social factors that we did not record.

### 4.3. Clinical Heterogeneity of Polypathology

Polypathology is not a homogeneous group. Latent class analysis separated seven phenotypes defined by combinations of categories recognizable in clinical practice, in line with the multimorbidity patterns described in other populations [33] and in Mexican adults [34]. We found no studies describing phenotypes within the polypathological population defined by the criteria of the Integrated Care Process. Because 68.4% of patients met exactly two categories, most classes reproduce the most frequent category pairs, and the osteoarticular class corresponds to all patients with category H, so a table of category combinations would describe most patients similarly. The latent class model adds a reproducible, probability-based assignment of the 31.6% of patients with three or more categories and a measure of classification uncertainty, but its value is mainly descriptive, as it summarizes the differences in age, sex, etiology, and care burden between groups.

The most frequent phenotype, the renal-diabetic, brought together chronic kidney disease and diabetes with microvascular damage or peripheral artery disease in all its members, which is consistent with the prevalence of diabetes and its poor control in the country [3]. The second, the hepatic-hematologic, included mainly men with a mean age of 56 years with chronic liver disease, of alcohol-related cause in two of every three cases, and anemia due to digestive losses or active neoplasia. This profile matches Mexican epidemiology. Liver diseases were the fifth leading cause of death in México in 2023, with 40,052 deaths, 72.4% of them in men [35], and in a multicenter cohort from central México, alcohol-associated liver disease was the leading cause of cirrhosis in men and metabolic disease in women [36], the same sex distribution that we observed.

The phenotypes did not differ in mortality during the index admission but did differ in polypharmacy and prior hospitalizations, even after adjustment for the Charlson index. This result reinforces the interpretation in Section 4.2: whether analyzed by number of categories or by phenotypes, polypathology describes the burden of chronic care and not the risk of the acute episode. The phenotypes also suggest hypotheses for care. Cardiorenal and renal-diabetic patients, with more drugs and more prior admissions, might benefit from medication reconciliation and close follow-up after discharge, whereas the hepatic-hematologic phenotype, with fewer chronic drugs but repeated admissions, might require care oriented toward liver disease; these strategies were not evaluated in this study. These phenotypes are exploratory and should be validated in other cohorts.

The phenotypes also separated a readmission burden that polypathology, as a dichotomous variable, did not distinguish. The cardiorenal, hepatic-hematologic, and renal-diabetic phenotypes concentrated prior hospitalizations, readmissions during the period, and most polypathological patients younger than 50 years, whereas the neurological, cardiometabolic, and cardiorespiratory phenotypes, of older patients, had fewer readmissions than patients with multimorbidity without polypathology, the neurological phenotype partly because of its higher mortality during the index admission (20.3%). The difference in readmissions was at the limit of significance (*p* = 0.051 and 0.046), and the concentration of younger patients in these three phenotypes largely reflects their age profile.

### 4.4. Polypathology and In-Hospital Mortality

Polypathology was not conceived as a prognostic index but as a criterion for identifying patients who need a comprehensive model of care [8]; its prognostic instrument is the PROFUND index [10,11]. The relevant prognostic comparison is therefore that of PROFUND versus the Charlson index, not that of polypathology versus the Charlson index. We evaluated mortality to answer a more limited question, namely whether polypathological status adds information to that of the Charlson index, and we found no clear evidence that it does, although the confidence intervals do not exclude a moderate association. The original series did find a threefold higher mortality [9], but it compared polypathological patients with the remaining Internal Medicine patients who were neither polypathological nor palliative, a more heterogeneous group than ours. Our comparison group also had multimorbidity, which poses a more demanding question. The crude difference we observed (13.5% versus 10.8%) was not significant and was attenuated after adjustment for age, sex, and the Charlson index. The inverse association between the number of categories and death when the Charlson index is in the model (OR 0.74; *p* = 0.018), and the inverted U-shaped pattern across categories, probably reflect adjustment for an index that shares much of its content with the categories, so that patients with the same mortality-weighted comorbidity burden are compared, and the small number of patients with four or more categories (n = 29), rather than a protective effect. Results were consistent when death during any admission of the period was analyzed with adjustment for follow-up time.

Death during an admission depends mainly on the acute reason for hospitalization and its severity, variables that neither polypathology nor the Charlson index captures. The PROFUND index, designed for the prognosis of these patients, includes functional, social, and laboratory variables and was validated for mortality at 12 months and at four years, not during admission [10,11]. In an Argentine cohort, PROFUND did discriminate in-hospital mortality (AUC 0.76), although in only 111 patients [12]. Therefore, our results do not contradict the medium-term prognostic value of polypathology, which we could not assess. The subanalysis of patients with a recorded Katz index are compatible with this interpretation, since functional dependence showed a stronger, although nonsignificant, association with death than polypathology, suggesting that the functional component, which PROFUND does incorporate, may weigh more in prognosis than the number of categories.

The original Charlson index also discriminated death during admission poorly (AUC 0.57; Table S6), which was expected for an index derived to predict one-year mortality [14]. In a Spanish hospital, a model combining the Charlson index with age reached an AUC of 0.71 for in-hospital mortality in an older population [37], and in the cohort in which the PROFUND index was developed, the Charlson index had an AUC of 0.59 for 12-month mortality [10]. Its age-adjusted version discriminated almost as well as age alone, and combining it with age in the same model counts age twice (OR of 2.26 for the age-adjusted Charlson index ≥ 4 in our data) [17]; it is therefore advisable to state which version of the index is used.

### 4.5. Other Findings

Male sex was associated with death during any admission of the period (OR 1.66; 1.19–2.32), but its association with death during the index admission was weaker and not significant (OR 1.37; 0.94–1.98). Men were younger and had a slightly higher Charlson index (mean 3.5 versus 3.0). We have no variables that explain this difference; we report it disaggregated, as recommended by the SAGER guidelines [38]. The inverse associations of moderate polypharmacy and of prior hospitalizations with mortality during the index admission should not be interpreted as protective effects. The inverse association of prior hospitalizations was largely explained by the timing of deaths, which in these patients occurred mainly during readmissions; the residual difference is compatible with selection of survivors of previous hospitalizations. The association with polypharmacy more likely reflects better prior access to care in patients with established chronic treatment, or incomplete recording of medication in the most severely ill patients. Studies that adjust in detail for comorbidity show that the association between the number of drugs and mortality is strongly affected by confounding [39].

### 4.6. Implications

For the internal medicine physician, the number of categories might help to identify patients who could benefit from medication reconciliation, close follow-up after discharge, and, when more categories accumulate, assessment for palliative care; these strategies were not evaluated in this study. Polypathology does not serve to estimate the risk of dying during admission, and neither does the Charlson index; for that purpose, variables of acute severity or validated instruments such as PROFUND are needed. From the perspective of the health system, finding this complexity profile in adults younger than in Spanish cohorts suggests that chronic care strategies aimed at working-age adults deserve evaluation.

### 4.7. Limitations and Strengths

This study has limitations. It is retrospective and single-center, in a hospital serving a population without social security coverage, so its results cannot be extrapolated to other health systems. Data were obtained without formal blinding to the outcome, although categories were recorded before the outcome was reviewed and were verified by a second investigator, and criteria that depend on documentation (NYHA class, mMRC grade, FEV1, or the duration of reduced kidney function) may have been underrecorded. The Katz index was missing in half of the patients, and those without a record had somewhat lower mortality, suggesting that it was omitted more often in less severely ill patients. Prior hospitalizations and readmissions were recorded only in the department itself, so they are underestimated; the census recorded a single type of discharge per patient, and the follow-up time for period outcomes varied according to the date of the first admission, which was adjusted for in the analyses. We could not calculate the PROFUND index, because several of its variables (Barthel index, main caregiver, and delirium during the previous hospitalization) are not systematically recorded. Finally, the phenotypes were obtained with an exploratory method in a single cohort, the BIC of the seven-class model was only 8 points lower than that of the six-class model, and the osteoarticular phenotype included only 30 patients.

The lack of systematic functional and cognitive assessment affected the classification in a predictable direction. Subcategories E2, E3, and H1 were assigned from the clinical description, so undocumented dependence or cognitive impairment was coded as not met; this probably underestimated categories E and H and, through them, the number of categories and the prevalence of polypathology. Because this error would mainly affect the most dependent patients, it is more likely to have attenuated than to have created the associations between the number of categories and care burden.

Among its strengths is the sample size, the largest published on polypathology in Latin America. A prospective multicenter study, with systematic functional and cognitive assessment and 12-month follow-up, is needed to confirm these findings in other health care settings in Mexico and Latin America and to validate PROFUND in the Mexican population.

## 5. Conclusions

In hospitalized adults without social security coverage in a Mexican public hospital, polypathology affected two of every three patients with multimorbidity and occurred in patients about 15 years younger than those in Spanish cohorts, although differences between cohorts limit this comparison. The number of categories was associated in a graded manner with polypharmacy and repeated hospitalizations at admission and with discharge at maximum therapeutic benefit, an association that persisted after adjustment for the Charlson index, although it was borderline for discharge at maximum benefit. Polypathology showed no clear association with mortality during the index admission once age and comorbidity burden were taken into account. Latent class analysis identified seven descriptive phenotypes that differed in care burden but not in mortality; one of them, made up mainly of middle-aged men with alcohol-related liver disease, reflects local epidemiology. The number of categories may help to identify patients who need a comprehensive model of care, but prospective multicenter validation is required before these findings are generalized to other health care settings in México or Latin America.

## Figures and Tables

**Figure 1:**
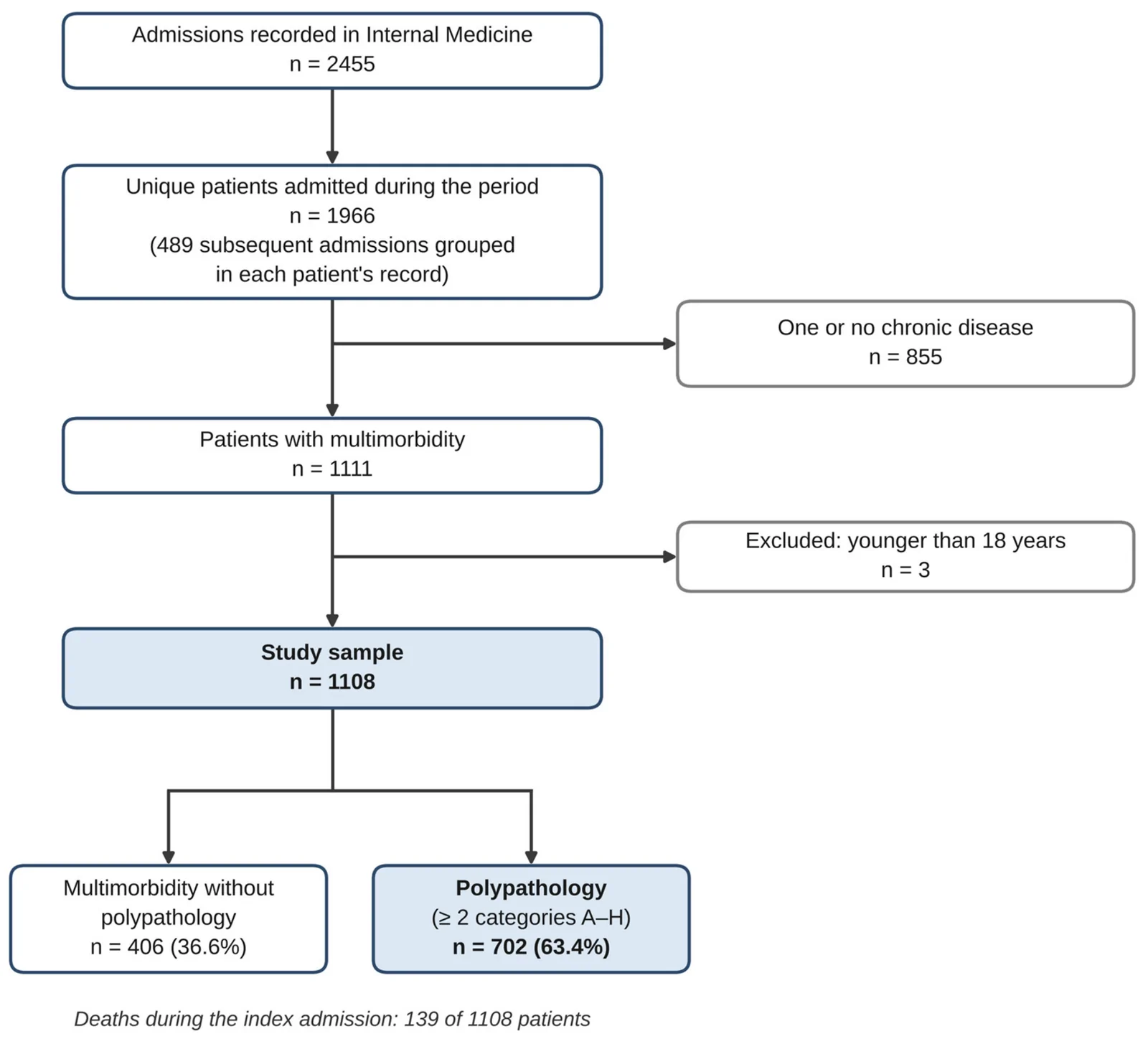
Flow diagram of patient selection.

**Figure 2:**
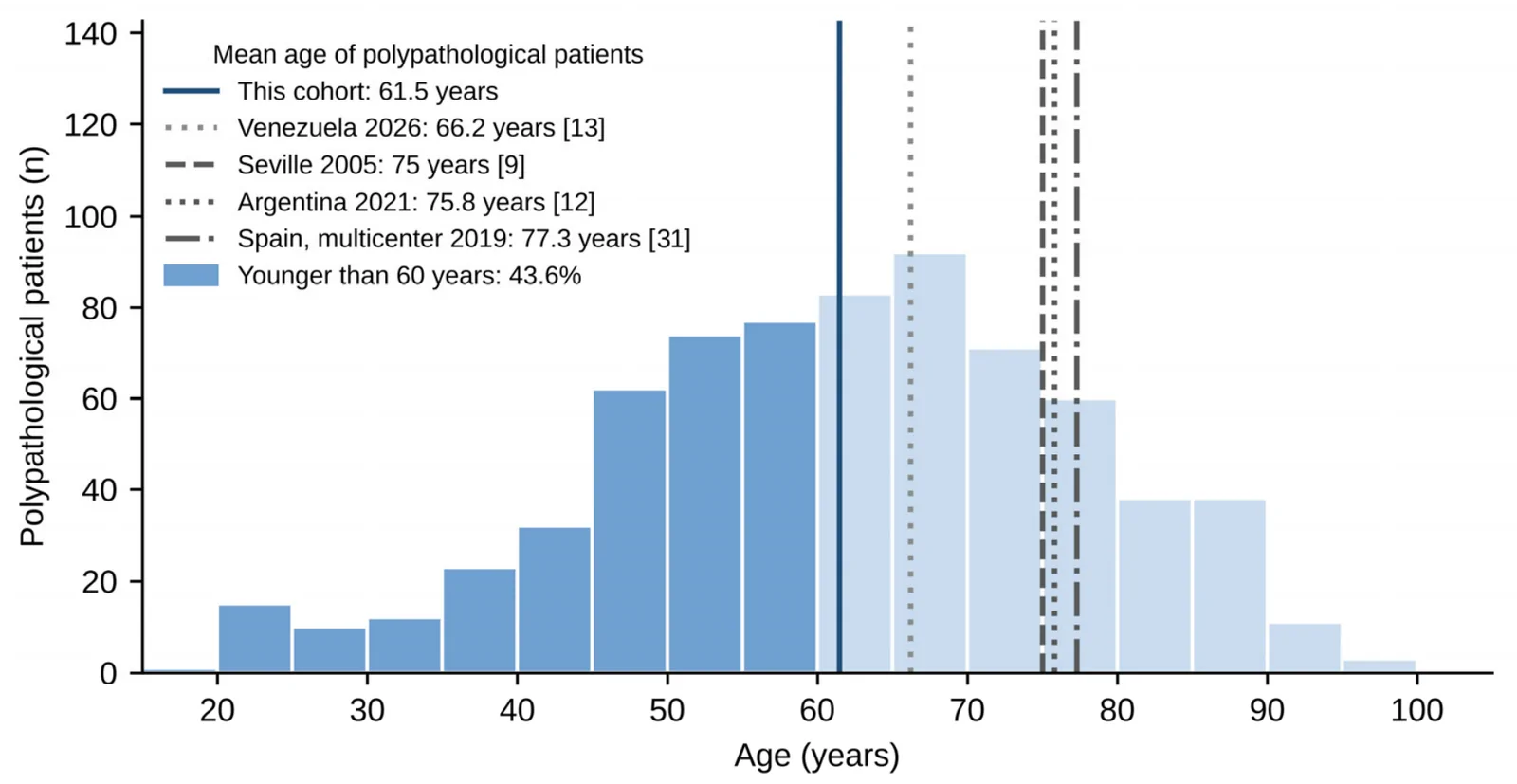
Age distribution of the 702 polypathological patients. Dark bars correspond to patients younger than 60 years. Vertical lines mark the mean age of this cohort and that of the published series from Seville [9], Argentina [12], Venezuela [13], and the Spanish multicenter cohort [31].

**Figure 3:**
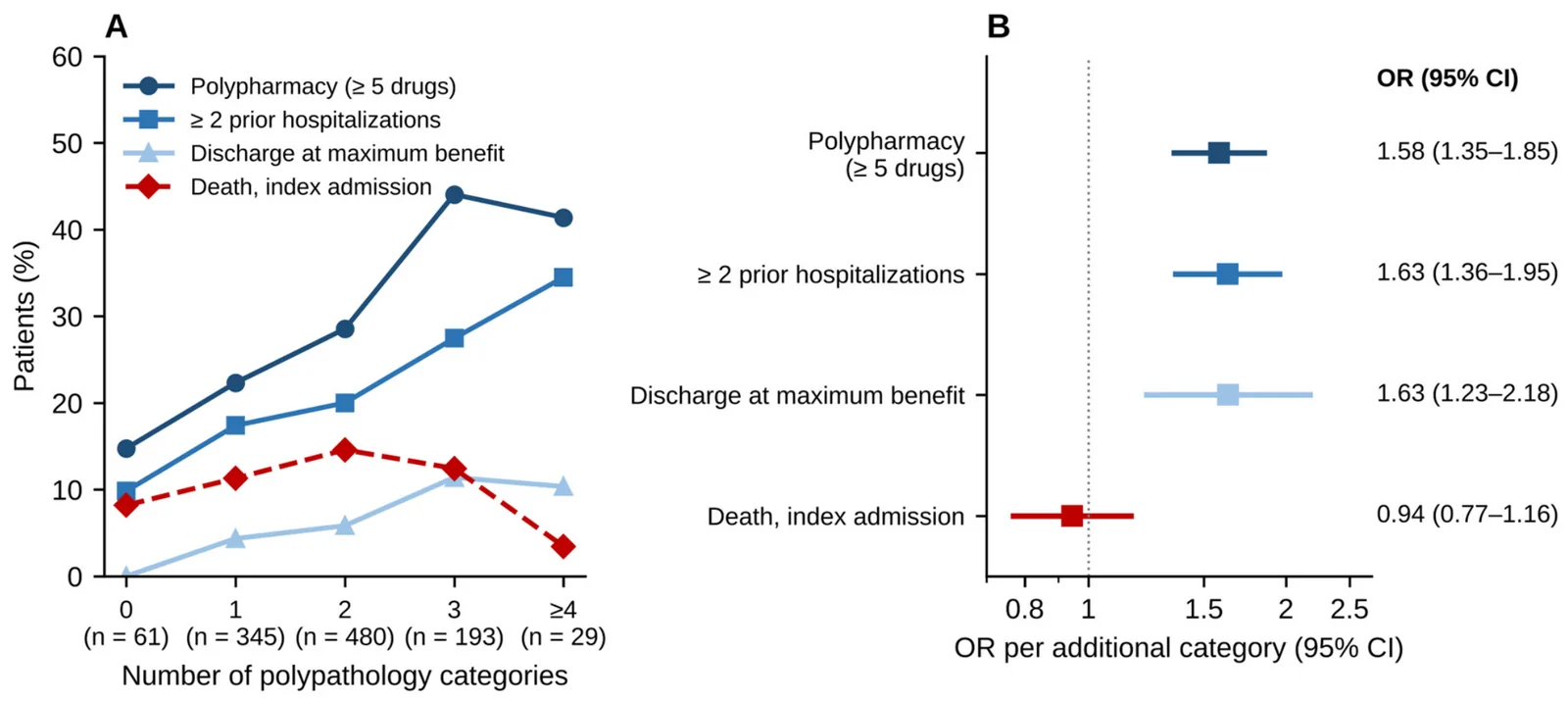
Markers of care complexity at admission, discharge at maximum benefit, and mortality during the index admission according to the number of polypathology categories. (**A**) Proportion of patients with each marker or outcome. (**B**) OR per additional category, with the number of categories as a continuous variable and adjustment for age (per decade) and sex (and for follow-up time in the case of discharge at maximum benefit); the dotted line marks OR = 1.

**Figure 4:**
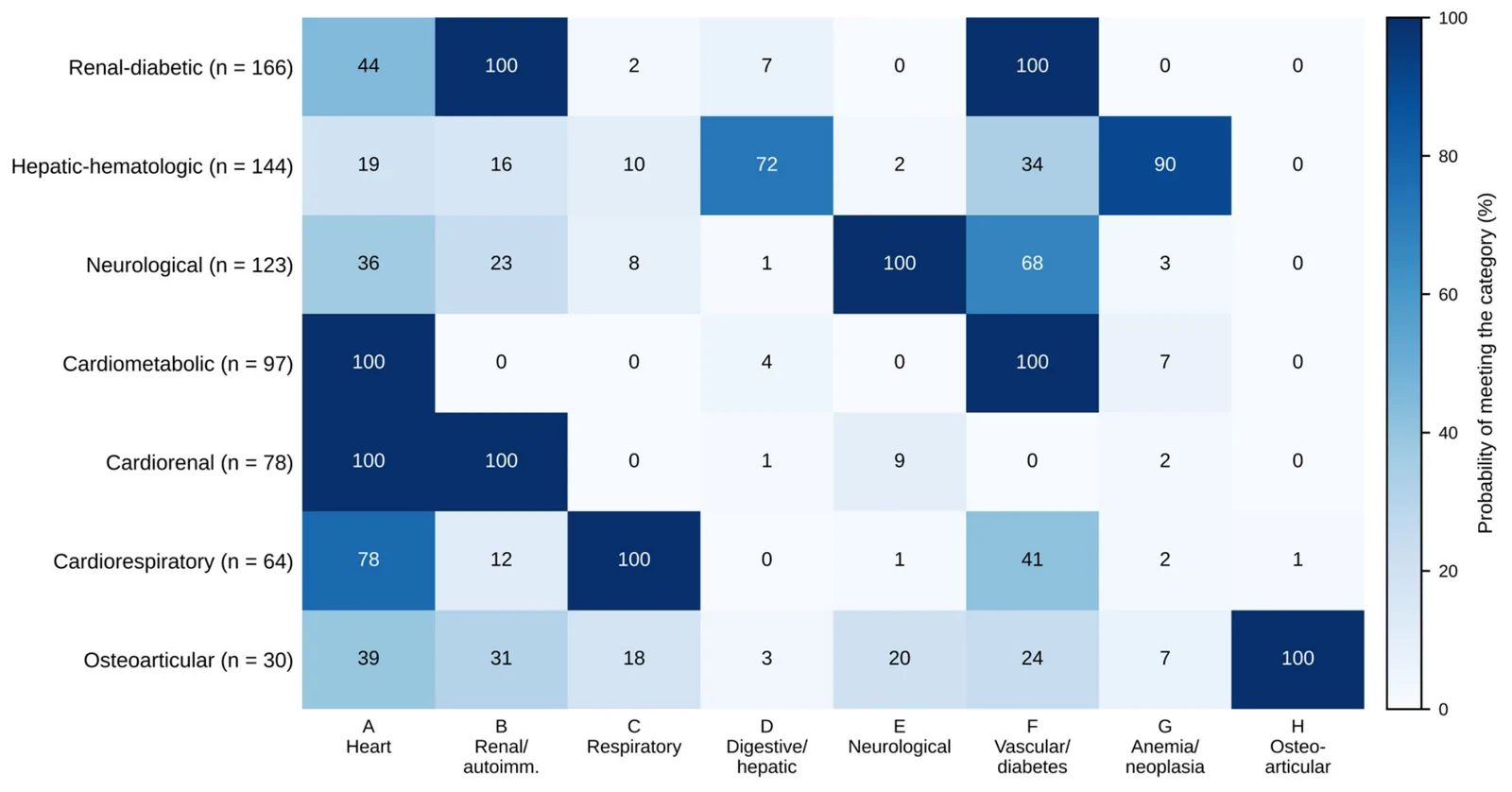
Polypathology phenotypes identified by latent class analysis. Each row is a phenotype and each column a polypathology category (A–H). Each cell shows the probability (%), estimated by the latent class model, that a patient of that phenotype meets the category: values close to 100% indicate that the category defines the phenotype, and values close to 0% that it is almost absent. The observed proportions, which differ from these probabilities by at most 4 percentage points, are shown in Table S7.

**Table 1:** Baseline characteristics according to the presence of polypathology.

Variable	Total *n* = 1108	Multimorbidity Without Polypathology *n* = 406	Polypathology *n* = 702	*p*
Age, years, mean ± SD	58.5 ± 17.6	53.3 ± 19.3	61.5 ± 15.8	<0.001
Age < 60 years, n (%)	547 (49.4)	241 (59.4)	306 (43.6)	<0.001
Male sex, n (%)	571 (51.5)	208 (51.2)	363 (51.7)	0.928
Original Charlson index, median (IQR)	3 (2–4)	2 (1–3)	4 (3–5)	<0.001
Age-adjusted Charlson index, median (IQR)	5 (3–6)	3 (2–5)	6 (4–7)	<0.001
Cardiovascular disease, n (%)	849 (76.6)	270 (66.5)	579 (82.5)	<0.001
Endocrine or metabolic disease, n (%)	653 (58.9)	166 (40.9)	487 (69.4)	<0.001
Respiratory disease, n (%)	127 (11.5)	28 (6.9)	99 (14.1)	<0.001
Chronic infectious disease, n (%)	88 (7.9)	51 (12.6)	37 (5.3)	<0.001
AIDS, n (%)	27 (2.4)	22 (5.4)	5 (0.7)	<0.001
Hematological or oncological disease, n (%)	183 (16.5)	82 (20.2)	101 (14.4)	0.015
Psychiatric disease, n (%)	49 (4.4)	27 (6.7)	22 (3.1)	0.010
Hemoglobin, g/dL, median (IQR)	11.1 (8.6–13.6)	11.8 (8.7–14.0)	10.9 (8.5–13.3)	0.025
Creatinine, mg/dL, median (IQR)	1.4 (0.8–3.8)	1.2 (0.8–3.2)	1.5 (0.9–4.1)	0.001
Polypharmacy (≥5 drugs), n (%)	320 (28.9)	86 (21.2)	234 (33.3)	<0.001
Severe polypharmacy (≥10 drugs), n (%)	28 (2.5)	4 (1.0)	24 (3.4)	0.022
≥2 hospitalizations in the previous year, n (%)	225 (20.3)	66 (16.3)	159 (22.6)	0.013
Dependence (Katz B–G), n (%) †	135 (24.5)	38 (18.7)	97 (28.0)	0.020
Underweight (BMI < 18.5 kg/m^2^), n (%)	125 (11.3)	66 (16.3)	59 (8.4)	<0.001
Obesity (BMI ≥ 30 kg/m^2^), n (%)	243 (21.9)	88 (21.7)	155 (22.1)	0.935

SD, standard deviation; IQR, interquartile range; BMI, body mass index. † Among the 550 patients with a recorded Katz index (203 without polypathology and 347 with polypathology). Disease groups correspond to the classification of chronic diseases used to define multimorbidity; AIDS corresponds to the Charlson index component. Hemoglobin and creatinine were taken from the first measurement within 24 h of admission.

**Table 2:** Hospital outcomes according to the presence of polypathology.

Outcome	Total *n* = 1108	Multimorbidity Without Polypathology *n* = 406	Polypathology *n* = 702	*p*
Length of stay, days, median (IQR)	5 (3–8)	5 (3–9)	5 (3–8)	0.644
Death during the index admission, n (%)	139 (12.5)	44 (10.8)	95 (13.5)	0.226
Death during any admission of the period, n (%)	182 (16.4)	57 (14.0)	125 (17.8)	0.122
≥1 readmission during the period, n (%)	247 (22.3)	91 (22.4)	156 (22.2)	>0.99
Type of last discharge of the period, n (%) ‡				0.002
Improvement	815 (73.6)	312 (76.8)	503 (71.7)	
Death	182 (16.4)	57 (14.0)	125 (17.8)	
Maximum therapeutic benefit	68 (6.1)	15 (3.7)	53 (7.5)	
Voluntary discharge	33 (3.0)	19 (4.7)	14 (2.0)	
Transfer	9 (0.8)	2 (0.5)	7 (1.0)	
Absconded	1 (0.1)	1 (0.2)	0 (0.0)	

IQR, interquartile range. ‡ The department census recorded a single type of discharge per patient: that of the last admission of the period. In the 861 patients without readmission, it corresponds to the index admission; in the 247 who were readmitted, to the last readmission. *p* for the overall comparison of discharge types by Fisher’s exact test (Monte Carlo simulation).

**Table 3:** Markers of care complexity and outcomes according to the number of polypathology categories (0 to ≥4). ORs correspond to each additional category.

Marker or Outcome	0 *n* = 61	1 *n* = 345	2 *n* = 480	3 *n* = 193	≥4 n = 29	OR (95% CI) §	*p*	OR with Charlson (95% CI) ¶	*p*
Markers at admission (cross-sectional)									
Polypharmacy (≥5 drugs)	9 (14.8)	77 (22.3)	137 (28.5)	85 (44.0)	12 (41.4)	1.58 (1.35–1.85)	<0.001	1.49 (1.23–1.81)	<0.001
≥2 prior hospitalizations	6 (9.8)	60 (17.4)	96 (20.0)	53 (27.5)	10 (34.5)	1.63 (1.36–1.95)	<0.001	1.34 (1.08–1.66)	0.007
Dependence (Katz B–G) †	7 (19.4)	31 (18.6)	59 (26.3)	33 (30.8)	5 (31.2)	1.17 (0.94–1.47)	0.166	1.05 (0.79–1.40)	0.735
Index admission									
Death	5 (8.2)	39 (11.3)	70 (14.6)	24 (12.4)	1 (3.4)	0.94 (0.77–1.16)	0.588	0.74 (0.58–0.95)	0.018
Period outcomes ‡									
Discharge at maximum benefit	0 (0.0)	15 (4.3)	28 (5.8)	22 (11.4)	3 (10.3)	1.63 (1.23–2.18)	<0.001	1.46 (1.03–2.08)	0.035
Death during any admission	6 (9.8)	51 (14.8)	91 (19.0)	31 (16.1)	3 (10.3)	0.97 (0.81–1.17)	0.770	0.79 (0.63–0.99)	0.044
Death or discharge at maximum benefit	6 (9.8)	66 (19.1)	119 (24.8)	53 (27.5)	6 (20.7)	1.15 (0.98–1.36)	0.093	0.94 (0.77–1.16)	0.574
≥1 readmission	11 (18.0)	80 (23.2)	107 (22.3)	44 (22.8)	5 (17.2)	1.03 (0.87–1.22)	0.704	0.94 (0.77–1.16)	0.575

Values are *n* (%). § Logistic regression with the number of categories as a continuous variable, adjusted for age (per decade) and sex. ¶ Same model with the original Charlson index (per point) as an additional covariate. † Only patients with a recorded Katz index. ‡ Last discharge of the period or any admission during the period; models additionally adjusted for the logarithm of follow-up time. Estimates with the number of categories as a categorical variable are shown in Table S8.

**Table 4:** Mortality during the index admission and proportion of patients with polypathology by strata of the original Charlson index.

Original Charlson Index	Patients, n (%)	Polypathology, n (%)	Deaths, n (%)
0–1	161 (14.5)	24 (14.9)	12 (7.5)
2–3	481 (43.4)	260 (54.1)	53 (11.0)
4–5	355 (32.0)	333 (93.8)	56 (15.8)
6–7	88 (7.9)	72 (81.8)	15 (17.0)
≥8	23 (2.1)	13 (56.5)	3 (13.0)

Percentages of polypathology and deaths were calculated within each stratum. OR for death per Charlson point adjusted for age (per decade) and sex: 1.13 (95% CI 1.02–1.24). The distribution according to the age-adjusted Charlson index is presented in Table S3.

**Table 5:** Multivariable logistic regression for death during the index admission (n = 1108; 139 deaths).

Variable	Primary Model, OR (95% CI)	*p*	Secondary Model, OR (95% CI)	*p*
Age (per decade)	1.32 (1.18–1.48)	<0.001	1.28 (1.14–1.44)	<0.001
Male sex	1.37 (0.94–1.98)	0.098	1.32 (0.91–1.92)	0.146
Original Charlson index (per point)	1.15 (1.03–1.28)	0.013	1.19 (1.07–1.33)	0.002
Polypathology	0.84 (0.54–1.30)	0.424	0.90 (0.58–1.40)	0.640
Polypharmacy 5–9 drugs (versus <5)	—		0.57 (0.36–0.93)	0.024
Polypharmacy ≥ 10 drugs (versus <5)	—		0.79 (0.26–2.38)	0.672
≥2 prior hospitalizations	—		0.30 (0.15–0.59)	<0.001

OR, odds ratio; CI, confidence interval. Primary model: age, sex, original Charlson index, and polypathology. Secondary model: additionally adjusted for polypharmacy and prior hospitalizations. models with all 1108 patients.

**Table 6:** Characteristics and outcomes of the polypathology phenotypes identified by latent class analysis (n = 702).

Phenotype	Dominant Categ.	*n* (%)	Age, Years	Men, *n* (%)	Charlson, Median (IQR)	Polypharmacy, *n* (%)	≥2 Prior Hosp., *n* (%)	Maximum Benefit, *n* (%)	Death (Index Admission), *n* (%)
Renal-diabetic	B + F	166 (23.6)	58.2 ± 13.6	87 (52.4)	4 (4–5)	69 (41.6)	57 (34.3)	18 (10.8)	23 (13.9)
Hepatic-hematologic	D + G	144 (20.5)	55.9 ± 14.0	95 (66.0)	4 (3–5)	28 (19.4)	40 (27.8)	14 (9.7)	13 (9.0)
Neurological	E + F	123 (17.5)	66.6 ± 14.9	64 (52.0)	4 (3–5)	35 (28.5)	12 (9.8)	8 (6.5)	25 (20.3)
Cardiometabolic	A + F	97 (13.8)	65.4 ± 12.8	43 (44.3)	3 (3–4)	33 (34.0)	10 (10.3)	2 (2.1)	13 (13.4)
Cardiorenal	A + B	78 (11.1)	55.7 ± 19.9	38 (48.7)	3 (3–4)	34 (43.6)	29 (37.2)	7 (9.0)	12 (15.4)
Cardiorespiratory	A + C	64 (9.1)	69.3 ± 15.6	25 (39.1)	3 (2–4)	27 (42.2)	7 (10.9)	2 (3.1)	6 (9.4)
Osteoarticular	H	30 (4.3)	70.8 ± 16.1	11 (36.7)	2 (2–3)	8 (26.7)	4 (13.3)	2 (6.7)	3 (10.0)
*p*			<0.001	0.002	<0.001	<0.001	<0.001	0.13	0.18

Age as mean ± SD. Dominant categ.: categories met by at least 60% of the members of the phenotype (Figure 4). Unadjusted *p* values: Kruskal–Wallis for continuous variables and Pearson’s χ^2^ for categorical variables. With likelihood ratio tests adjusted for age and sex, and for age, sex, and the original Charlson index, *p* values were <0.001 and <0.001 for polypharmacy and for prior hospitalizations, 0.049 and 0.10 for discharge at maximum benefit (with additional adjustment for follow-up time), and 0.23 and 0.26 for death during the index admission. Maximum benefit refers to the last discharge of the period. SD, standard deviation; hosp., hospitalizations; IQR, interquartile range.

## Data Availability

The data supporting this study are available on reasonable request from the corresponding author because of confidentiality restrictions on clinical information.

## Supplementary Materials

The following supporting information can be downloaded at: https://www.mdpi.com/article/10.3390/jcm15218722/s1, Table S1: Polypathology categories of the Integrated Care Process, 2018; Table S2: Polypathology categories: frequency, age, mortality during the index admission, and association with death; Table S3: Mortality during the index admission and proportion of patients with polypathology by strata of the age-adjusted Charlson index; Table S4: Characteristics of patients with and without a recorded Katz index; Table S5: Fit indices of the latent class models with the eight polypathology categories; Table S6: Discrimination for death during the index admission; Table S7: Distribution of the polypathology categories within each phenotype; Table S8: Mortality according to the number of polypathology categories as a categorical variable, and sensitivity analysis for death during any admission of the period; Table S9: Polypathology as a dichotomous variable: association with markers of care complexity and outcomes, and discrimination of the number of categories and of the original Charlson index; Figure S1: ROC curves for death during the index admission.

## Abbreviations

The following abbreviations are used in this manuscript:

AIDS	Acquired immunodeficiency syndrome
AUC	Area under the receiver operating characteristic curve
BIC	Bayesian information criterion
BMI	Body mass index
CI	Confidence interval
COPD	Chronic obstructive pulmonary disease
FEV1	Forced expiratory volume in the first second
IQR	Interquartile range
mMRC	Modified Medical Research Council
NYHA	New York Heart Association
OR	Odds ratio
PAI	Proceso Asistencial Integrado (Integrated Care Process)
PROFUND	Prognostic index for polypathological patients
ROC	Receiver operating characteristic
SAGER	Sex and Gender Equity in Research
SD	Standard deviation
STROBE	Strengthening the Reporting of Observational Studies in Epidemiology
