# Supplementary material for: Polypathology as a Marker of Care Complexity in Hospitalized Adults with Multimorbidity in México
Source: J Clin Med. 2026 Nov 8;15(21):8722. doi: 10.3390/jcm15218722 (PMC13764155; doi:10.3390/jcm15218722)
Supplement: Supplementary file 1 [file jcm-15-08722-s001.zip › jcm-4656097-supplementary.pdf]

Supplementary materials

Polypathology as a marker of care complexity in hospitalized adults with multimorbidity in México

Table S1. Polypathology categories of the Integrated Care Process, 2018 [8].

Category	Subcategory	Criterion
A	A1	Heart failure with NYHA class II dyspnea in a stable situation
A	A2	Ischemic heart disease
B	B1	Vasculitis and systemic autoimmune diseases
B	B2	Chronic kidney disease (glomerular filtration rate < 60 mL/min or albuminuria > 30 mg/g)
C	C1	Chronic respiratory disease with mMRC grade 2 dyspnea, FEV1 < 70%, or SatO2 ≤ 90%
D	D1	Inflammatory bowel disease
D	D2	Chronic liver disease with hepatocellular failure or portal hypertension
E	E1	Stroke
E	E2	Neurological disease with permanent motor deficit limiting basic activities
E	E3	Permanent cognitive impairment, at least moderate
F	F1	Symptomatic peripheral artery disease
F	F2	Diabetes with proliferative retinopathy or symptomatic neuropathy
G	G1	Chronic anemia due to digestive losses or acquired hemopathy not amenable to curative treatment (Hb < 10 g/dL in two measurements more than three months apart)
G	G2	Active solid or hematological neoplasm not amenable to curative treatment
H	H1	Chronic osteoarticular disease limiting bed-to-chair transfer
H	H2	Osteoporotic hip fracture

Patients with two or more categories were classified as polypathological. NYHA, New York Heart Association; mMRC, modified Medical Research Council scale; FEV1, forced expiratory volume in the first second; SatO2, arterial oxygen saturation; Hb, hemoglobin.

Table S2. Polypathology categories: frequency, age, mortality during the index admission, and association with death.

**Category**	**n (% of PP)**	**Mean age, years**	Deaths (index admission), **n (%)**	**Age- and sex-adjusted OR (95% CI)**	**p**
A	376 (53.6)	64.4	53 (14.1)	0.99 (0.63–1.55)	0.975
B	309 (44.0)	58.6	38 (12.3)	0.90 (0.57–1.40)	0.636
C	98 (14.0)	68.7	8 (8.2)	0.45 (0.21–0.97)	0.043
D	126 (17.9)	54.3	11 (8.7)	0.62 (0.31–1.22)	0.165
E	141 (20.1)	66.8	28 (19.9)	1.65 (1.00–2.70)	0.049
F	428 (61.0)	62.3	59 (13.8)	1.05 (0.67–1.65)	0.822
G	150 (21.4)	57.4	16 (10.7)	0.75 (0.42–1.34)	0.335
H	30 (4.3)	70.8	3 (10.0)	0.60 (0.18–2.06)	0.420

PP, polypathology. A patient may meet several categories. Comparison with polypathological patients without that category. None of the associations would remain significant after correction for eight comparisons (Bonferroni threshold p < 0.006).

Table S3. Mortality during the index admission and proportion of patients with polypathology by strata of the age-adjusted Charlson index.

**Age-adjusted Charlson index**	**Patients, n (%)**	**Polypathology, n (%)**	**Deaths, n (%)**
0–1	61 (5.5)	4 (6.6)	1 (1.6)
2–3	261 (23.6)	90 (34.5)	16 (6.1)
4–5	350 (31.6)	242 (69.1)	44 (12.6)
6–7	300 (27.1)	243 (81.0)	54 (18.0)
≥ 8	136 (12.3)	123 (90.4)	24 (17.6)

Percentages of polypathology and deaths were calculated within each stratum. The adjusted index adds one point for each decade from age 50 years; the steeper mortality gradient than in Table 4 largely reflects the age component.

Table S4. Characteristics of patients with and without a recorded Katz index.

**Variable**	**Katz recorded (*n* = 550)**	**Katz not recorded (*n* = 558)**	***p***
Age, years, mean ± SD	63.5 ± 16.8	53.5 ± 16.9	< 0.001
Male sex, n (%)	257 (46.7)	314 (56.3)	0.002
Original Charlson index, median (IQR)	3 (2–4)	3 (2–4)	0.246
Polypathology, n (%)	347 (63.1)	355 (63.6)	0.904
Number of categories, median (IQR)	2 (1–2)	2 (1–2)	0.587
Polypharmacy (≥ 5 drugs), n (%)	170 (30.9)	150 (26.9)	0.158
≥ 2 prior hospitalizations, n (%)	99 (18.0)	126 (22.6)	0.069
Length of stay, days, median (IQR)	5 (3–8)	5 (3–9)	0.029
Discharge at maximum benefit, n (%) †	39 (7.1)	29 (5.2)	0.235
Death during the index admission, n (%)	80 (14.5)	59 (10.6)	0.057

Mortality model in the 550 patients with a recorded Katz index (80 deaths during the index admission), adjusted ORs (95% CI): age per decade 1.23 (1.04–1.45); male sex 1.32 (0.81–2.15); original Charlson index per point 1.22 (1.06–1.41); functional dependence (Katz B–G) 1.55 (0.90–2.65); polypathology 0.74 (0.41–1.33). † Last discharge of the period. SD, standard deviation; IQR, interquartile range.

Table S5. Fit indices of the latent class models with the eight polypathology categories.

**Classes**	**Log-likelihood**	**BIC**	**AIC**	**Entropy**	**Smallest class, n (%)**	**Minimum mean posterior probability**
2	−2,645.2	5,401.9	5,324.5	0.94	138 (19.7)	0.94
3	−2,584.7	5,339.8	5,221.4	0.95	122 (17.4)	0.93
4	−2,529.6	5,288.6	5,129.2	0.94	127 (18.1)	0.92
5	−2,492.0	5,272.3	5,071.9	0.94	70 (10.0)	0.90
6	−2,457.0	5,261.4	5,020.1	0.92	78 (11.1)	0.87
**7**	**−2,423.4**	**5,253.2**	**4,970.9**	**0.97**	**30 (4.3)**	**0.95**
8	−2,404.2	5,273.7	4,950.4	0.95	28 (4.0)	0.86

The seven-class model (in bold), with the lowest BIC, was chosen. Each model was estimated with 300 sets of random starting values (poLCA 1.6.0.2 in R). AIC, Akaike information criterion; BIC, Bayesian information criterion.

Table S6. Discrimination for death during the index admission.

**Predictor or model**	**AUC (95% CI)**	**AUC difference (95% CI)**	**p (DeLong)**
Polypathology (yes/no)	0.53 (0.49–0.57)		
Number of polypathology categories	0.52 (0.47–0.56)		
Original Charlson index	0.57 (0.53–0.62)		
Age-adjusted Charlson index	0.63 (0.59–0.68)		
Age	0.62 (0.58–0.67)		
Model 1: age + sex	0.633	reference	
Model 2: model 1 + original Charlson	0.652	0.019 (−0.004 to 0.041) versus model 1	0.108
Model 3: model 2 + polypathology	0.654	0.003 (−0.006 to 0.011) versus model 2	0.522
Model 4: model 2 + number of categories	0.664	0.012 (−0.008 to 0.032) versus model 2	0.226

AUC, area under the ROC curve; confidence intervals by the DeLong method. Adding polypathology to model 2: integrated discrimination improvement 0.0008 (bootstrap 95% CI −0.00005 to 0.0088) and continuous net reclassification index −0.14 (bootstrap 95% CI −0.22 to 0.30). The bootstrap distribution of the net reclassification index is bimodal: when a binary predictor with a near-null effect is added, the sign of its coefficient changes between replicates (negative in 76% of them), and the index takes values of opposite sign accordingly, so its percentile interval is wide and includes the point estimate only near one end. Calibration (Hosmer–Lemeshow): model 2, p = 0.48; model 3, p = 0.38.

Table S7. Distribution of the polypathology categories within each phenotype.

**Phenotype**	**n**	**A**	**B**	**C**	**D**	**E**	**F**	**G**	**H**	**Mean no. of categories**
Renal-diabetic	166	72 (43.4)	166 (100.0)	3 (1.8)	12 (7.2)	0 (0.0)	166 (100.0)	0 (0.0)	0 (0.0)	2.52
Hepatic-hematologic	144	24 (16.7)	21 (14.6)	14 (9.7)	107 (74.3)	2 (1.4)	46 (31.9)	132 (91.7)	0 (0.0)	2.40
Neurological	123	42 (34.1)	26 (21.1)	11 (8.9)	1 (0.8)	123 (100.0)	84 (68.3)	5 (4.1)	0 (0.0)	2.37
Cardiometabolic	97	97 (100.0)	0 (0.0)	0 (0.0)	4 (4.1)	0 (0.0)	97 (100.0)	8 (8.2)	0 (0.0)	2.12
Cardiorenal	78	78 (100.0)	78 (100.0)	0 (0.0)	1 (1.3)	10 (12.8)	0 (0.0)	2 (2.6)	0 (0.0)	2.17
Cardiorespiratory	64	51 (79.7)	9 (14.1)	64 (100.0)	0 (0.0)	0 (0.0)	28 (43.8)	1 (1.6)	0 (0.0)	2.39
Osteoarticular	30	12 (40.0)	9 (30.0)	6 (20.0)	1 (3.3)	6 (20.0)	7 (23.3)	2 (6.7)	30 (100.0)	2.43
**All polypathological**	**702**	**376 (53.6)**	**309 (44.0)**	**98 (14.0)**	**126 (17.9)**	**141 (20.1)**	**428 (61.0)**	**150 (21.4)**	**30 (4.3)**	**2.36**

Values are n (%) of the members of each phenotype who meet each category; categories met by at least 60% define the phenotype (Table 6). Figure 4 shows the corresponding probabilities estimated by the latent class model, which differ from these observed proportions by at most 4 percentage points. A, heart disease; B, chronic kidney disease or systemic autoimmune disease; C, chronic respiratory disease; D, chronic liver disease or inflammatory bowel disease; E, neurological disease; F, peripheral artery disease or diabetes with microvascular damage; G, chronic anemia or active neoplasia; H, disabling osteoarticular disease or hip fracture.

Table S8. Mortality according to the number of polypathology categories as a categorical variable, and sensitivity analysis for death during any admission of the period.

	**0 (*n* = 61)**	**1 (*n* = 345)**	**2 (*n* = 480)**	**3 (*n* = 193)**	**≥ 4 (*n* = 29)**
Death during the index admission (139 deaths)					
Deaths, n (%)	5 (8.2)	39 (11.3)	70 (14.6)	24 (12.4)	1 (3.4)
OR (95% CI), age and sex	1 (ref.)	1.50 (0.56–4.00)	1.72 (0.66–4.50)	1.30 (0.47–3.60)	0.31 (0.03–2.83)
OR (95% CI), age, sex, and Charlson	1 (ref.)	1.26 (0.47–3.40)	1.13 (0.42–3.03)	0.63 (0.21–1.89)	0.11 (0.01–1.12)
Death during any admission of the period (182 deaths)					
Deaths, n (%)	6 (9.8)	51 (14.8)	91 (19.0)	31 (16.1)	3 (10.3)
OR (95% CI), age, sex, and follow-up	1 (ref.)	1.61 (0.65–3.98)	1.88 (0.77–4.55)	1.37 (0.53–3.52)	0.77 (0.18–3.41)
OR (95% CI), age, sex, follow-up, and Charlson	1 (ref.)	1.40 (0.56–3.50)	1.32 (0.53–3.29)	0.75 (0.27–2.06)	0.34 (0.07–1.63)

Number of categories entered as a categorical variable, with no categories as the reference; quadratic term for the number of categories, p = 0.029 for death during the index admission and p = 0.031 for death during any admission. Follow-up: logarithm of the months from the index admission to the end of the period. For death during any admission of the period, adjusted for age, sex, the original Charlson index, and follow-up time, the OR for polypathology was 0.90 (0.60–1.33) and that for male sex 1.66 (1.19–2.32); the OR per additional category was 0.97 (0.81–1.17), and 0.79 (0.63–0.99) with the Charlson index. In the secondary model, which added polypharmacy and prior hospitalizations, the ORs were 0.95 (0.64–1.41) for polypathology, 1.60 (1.14–2.25) for male sex, and 0.88 (0.57–1.38) for two or more prior hospitalizations.

Table S9. Polypathology as a dichotomous variable: association with markers of care complexity and outcomes, and discrimination of the number of categories and of the original Charlson index.

	**Events, *n* (%)**	**OR for polypathology (95% CI), age and sex**	***p***	**OR for polypathology (95% CI), plus Charlson**	***p***	**AUC (95% CI), number of categories**	**AUC (95% CI), Charlson index**
Polypharmacy (≥ 5 drugs)	320 (28.9)	1.89 (1.41–2.53)	< 0.001	1.55 (1.12–2.16)	0.009	0.60 (0.57–0.64)	0.59 (0.55–0.62)
≥ 2 prior hospitalizations	225 (20.3)	1.97 (1.40–2.76)	< 0.001	1.40 (0.96–2.03)	0.078	0.57 (0.53–0.61)	0.62 (0.58–0.66)
Discharge at maximum benefit ‡	68 (6.1)	1.99 (1.09–3.61)	0.025	1.51 (0.79–2.91)	0.212	0.62 (0.56–0.69)	0.60 (0.53–0.67)
Death during the index admission	139 (12.5)	1.08 (0.73–1.60)	0.691	0.84 (0.54–1.30)	0.424	0.52 (0.47–0.56)	0.57 (0.53–0.62)

Logistic regression adjusted for age (per decade) and sex, and additionally for the original Charlson index (per point). ‡ Last discharge of the period; models additionally adjusted for the logarithm of follow-up time. AUC, area under the ROC curve, with 95% confidence intervals by the DeLong method; the number of categories (0 to 8) and the Charlson index were entered as single predictors. The ORs per additional category are shown in Table 3 of the main text.

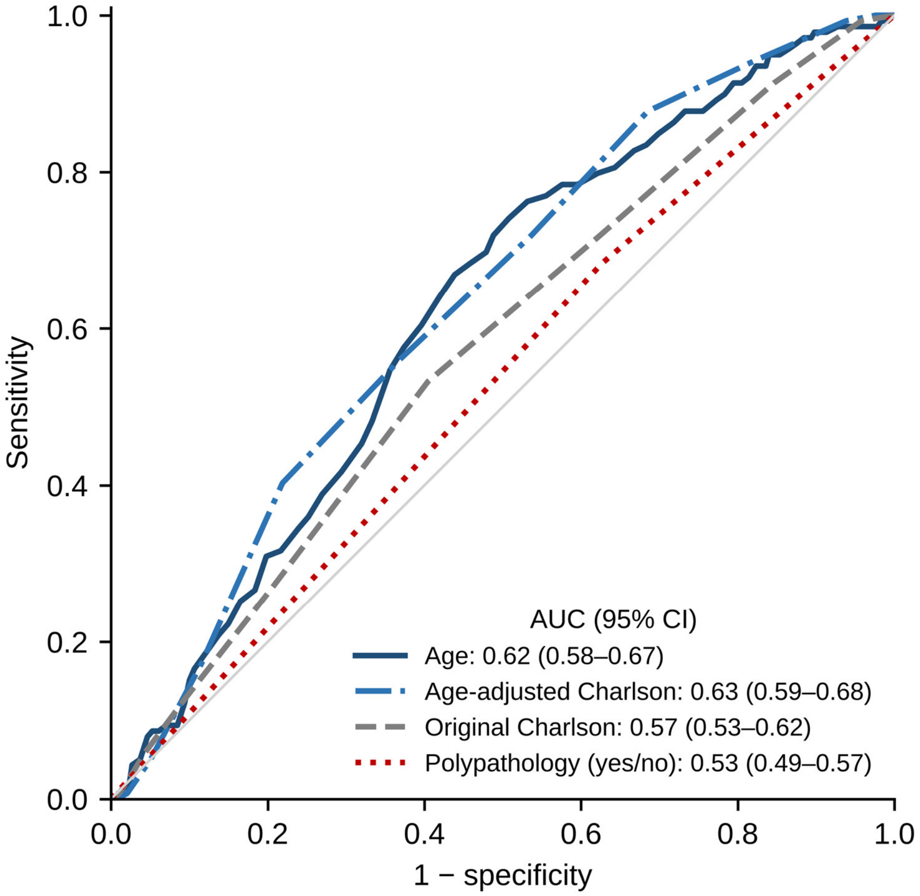

Figure S1. ROC curves for death during the index admission (n = 1,108; 139 deaths). Age, the age-adjusted Charlson index, the original Charlson index, and polypathology are shown as single predictors, with their AUC and 95% confidence interval by the DeLong method. The diagonal represents no discrimination.
